# Prevalence of neuroleptic malignant syndrome in 672 consecutive male in-patients

**DOI:** 10.4103/0019-5545.55089

**Published:** 2009

**Authors:** Pradyot Sarkar, Chandrashekhar Natarajan, Neeta Gode

**Affiliations:** Department of Psychiatry, Command Hospital, Alipore, Kolkata, West Bengal, India; 1 Military Hospital, Jhansi, UP, India

**Keywords:** Haloperidol, manic episode, neuroleptics, neuroleptic malignant syndrome

## Abstract

**Background:**

Neuroleptic malignant syndrome (NMS) is the most serious and potentially fatal adverse effect of neuroleptic medications. Although it occurs most frequently with conventional antipsychotic medicines, it may occur with newer antipsychotic medicines also. So far, there is no study available about the prevalence of this condition in the Indian population.

**Aim:**

To find the prevalence of NMS in the Indian population

**Materials and Methods:**

A total of 672 consecutive male patients who were hospitalized with psychotic breakdowns and treated with neuroleptics were screened for evidence of the development of NMS, clinically, and supported by laboratory investigations.

**Results:**

Three patients, all suffering from manic episode, developed NMS within few days of initiation of neuroleptic medicines. Haloperidol injection was given in all three cases

**Conclusion:**

The prevalence of NMS was 0.45%. All were young males, suffering from manic episode with psychotic symptoms and managed with oral and parentral neuroleptic medicines.

## INTRODUCTION

Of the various adverse effects of neuroleptic medicines, neuroleptic malignant syndrome (NMS) is the most serious and potentially fatal.[1,2] Although it occurs most frequently with the use of high potency conventional antipsychotic medicines, this condition may accompany treatment with any antipsychotic agent, including the newer atypical antipsychotics. Patients may have marked muscle rigidity, elevated temperature, diaphoresis, dysphagia, incontinence, altered sensorium, mutism, elevated or labile blood pressure, elevated white blood cell counts, and elevated serum creatinine phosphokinase (CPK).[2,3] The condition has been reported since 1960 and one should not automatically diagnose extrapyramidal adverse effects and fever as NMS. The estimates of 1-year prevalence of NMS in individuals exposed to antipsychotic medications range from 0.02 to 2.4% and mortality rates reported are in the range of 10-20%. No clear evidence implicates any particular typical neuroleptic agent as more or less likely to cause NMS; atypical neuroleptics (e.g. clozapine and risperidone) have also been reported to be associated with NMS. This syndrome tends to develop when neuroleptic treatment is initiated or the dosage is increased, particularly when the dose is high or given parenteral.[4]

Though antipsychotic medicines are regularly used in psychiatric practice, one rarely comes across a case of NMS. In the search done by us, we could not find any Indian study on the prevalence of NMS. Few foreign authors have reported high prevalence, as high as in 2.4% of patients treated with antipsychotic medicines.[4,5] Such wide variation of reported prevalence and lack of Indian studies had generated interest in screening of all patients exposed to antipsychotic medicines for evidence of NMS.

## MATERIALS AND METHODS

A total of 672 consecutive male patients, who were hospitalized in the psychiatry department of two general hospitals of India with psychotic breakdowns and managed with antipsychotics medicines, were included in the study. This study was a hospital- based prospective study conducted during the years 2001-2006. All the subjects were males, above the age of 18 years. They were diagnosed as per Diagnostic criteria for research of ICD-10 Classification of Mental and Behavioural Disorders,[7] such as schizophrenia, persistent delusional disorders, acute and transient psychotic disorders, other nonorganic psychotic disorders, and mood disorders. All patients got at least 6 weeks of antipsychotic treatment (typical and/or atypical) as in-patients and subsequently managed as out-patients. During their in-patient treatment, they were closely monitored for the development of any sign/symptoms suggestive of NMS. Diagnosis of NMS was done as per DSM criteria. These were diaphoresis, tachycardia, elevated or labile blood pressure, dysphagia, incontinence, tremor, rigidity, altered sensorium, mutism, leukocytosis, and laboratory evidence of muscle injury, i.e. elevated CPK, (normal value 15-130 U/L). The dose of antipsychotic medicines during the in-patient treatment was of standard adult therapeutic dose ranging between 500 and 1000 mg chlorpromazine equivalents.[8]

## RESULTS

A majority of the patients were young adults. In the age group of 18-35 yrs, there were 472 patients and in the age group of 36 years and above there were 200 patients. Patients were managed with typical and atypical antipsychotic medicines. Of these, 253 patients received only typical antipsychotics, whereas 321 received only atypical and 49 patients received a combination of typical and atypical antipsychotics. Patients who received combination treatment were initially started with an atypical antipsychotic and within few days injectable typical antipsychotics were added for better behavioural control. Eighty-three patients needed injectable neuroleptics (haloperidol), 5-10 mg IM SOS, for initial few days of hospitalization for behavioral control. Of the 672 patients, only 3 developed NMS. Among the 672 patients, 467 suffered from a psychotic illness (schizophrenia 251, persistent delusional disorder 27, acute and transient psychotic disorder 74 and other non-organic psychotic disorder 115) and the remaining 205 patients suffered from a manic episode/bipolar affective disorder, currently having a manic episode.

### Case 1

An 18 yrs old male had a manic episode of abrupt onset in summer of 2003. He was hospitalized within few days. Before hospitalization, he was administered with tab chlorpromazine (100 mg) twice daily for 3 days. On examination, he was found to be tall, thin built man, weighing 58 kg, who was talking continuously, often singing, dancing and was easily getting distracted by external cues. He was very agitated, getting violent, and was mildly dehydrated. His pulse rate was 90/min, blood pressure 110/70 mmHg and body temperature 98°F. He was given inj haloperidol (10 mg) along with inj promethazine (25 mg) IM in the morning on the day of admission and even required to be physically restrained. The patient slept for few hours after that and in the evening developed acute dystonia of neck, which was managed with inj promethazine (25 mg) IM. The patient was observed to be having marked rigidity the next morning. Treatment was started with tab ziprasidone (40 mg) twice daily and tab trihexyphenidyl (2 mg) twice daily. On the third morning of hospitalization, he was almost mute, having marked rigidity and persistent dystonic movements involving trunk, neck, and limbs. His pulse rate was 106/min, BP 140/100 mmHg, body temperature 103°F and was having diaphoresis. He was not accepting food and was little drowsy. NMS was clinically suspected; hence, all medicines were withheld and he was shifted to intensive care unit. CPK was 1450 IU/L. He was managed with tab diazepam (5mg), twice daily, bromocriptine (2.5 mg) thrice daily, trihexyphenidyl (2 mg) twice daily, and other symptomatic treatment. The next day, he improved and CPK level was 810 IU/L. His rigidity and fever resolved completely by fourth day and CPK level came to normal on the fifth day. Other laboratory parameters and brain imaging were normal. He then showed emergence of manic features for which tab lithium and clonzepam were started from the fifth day of treatment. Clonazepam was discontinued after 1 week and lithium continued in therapeutic range. He was managed as in-patient for 2 months [Table 1].

**Table:** Comparison of neuroleptic malignant syndrome in three patients

	Case I	Case II	Case III
Age (years)	18	19	23
Diagnosis	Manic episode with psychotic symptoms	Manic episode with psychotic symptoms	Manic episode with psychotic symptoms
Medicines used prior to development of NMS	Tab. chlorpromazine 100 mg, 1 BD × 3 days, inj. haloperidol 10 mg, inj. promethazine 25 mg + 25 mg, tab. ziprasidone 40 mg, 1 BD × 2 days, tab. trihexyphenidyl 2 mg, 1 BD × 2 days	Tab. chlorpromazine 100 mg, 1 BD × 5 days, tab. haloperidol 5 mg, 1 BD × 5 days, tab. promethazine 25 mg, 1 TDS × 5 days, inj. haloperidol 10mg and inj. promethazine 25 mg IM twice	Tab. haloperidol 5 mg, 1 BD × 3 days then 1 TDS × 5 days, tab. trihexyphenidyl 2 mg, 1 TDS × 5 days, tab. sodium valproate 200 mg 2 TDS × 5 days, inj. haloperidol 5 mg IM on four occasions in the first 3 days
Development of NMS	On 6^th^ day	On 6^th^ day	On 5^th^ day
Agitation	+++	+++	++
Body temperature	103° F	99°F	100.3°F
BP (mmHg)	140/100	190/110	146/98
Diaphoresis	+++	+++	+++
Altered sensorium	+	+	+
Rigidity	+++	+++	+++
Incontinence	+	+	+
Dehydration	+	+	+
Muteness	+	+	+
Duration of NMS	3 days	7 days	2 days
Catatonic features	-	+	-
CPK in U/L (normal 15-130 U/L)	1450	4840	964
TLC in mm^3^	10 800	12 000	10 900
Past history of neurological symptom or diseases	Nil	Nil	Nil
Family history of allergic or neurological illnesses	Nil	Nil	Nil

Tab.: Tablet; Inj.: Injection

### Case 2

The second case occurred in summer months in May 2004. This was a 19 yrs old male, tall and thin built, who developed a manic episode abruptly. He was talking excessively, using abusive language, greeting everybody repeatedly, boasting that he could make his team the best, delivering lectures, and commanding others in a loud voice. He was not sleeping, disobeying orders, and spending nights making plans for the improvement in his people and ate only few meals as he had no time to eat. He was very energetic despite not eating and sleeping. He was hospitalized within 2 days and for initial 5 days managed in a peripheral hospital with tab chlorpromazine (100 mg) twice daily, tab haloperidol (5 mg) twice daily, and tab promethazine (25 mg) thrice daily, and inj haloperidol (10 mg) IM with inj promethazine (25 mg) given on two occasions. When he reached the psychiatry center in the evening, he had features of mania. Initial examination revealed height: 178cm, weight: 65 kg, pulse rate: 88/min, BP: 150/90 mmHg, body temperature: 98.4°F, and profuse sweating. He was disheveled, distractible but confident and hyperactive. He was imitating interviewer's talk and actions. He had pressure of speech, flight of ideas, inflated self-esteem, irritable and elated affect, and was often uncooperative. After some time, he refused to be interviewed claiming himself to be a very important person. In the ward, he became aggressive and needed to be physically restrained and inj haloperidol 5 mg and inj promethazine 25 mg IM were given at 2300 hrs. He passed urine in bed that night and did not take breakfast next morning. He was found to be disheveled, hyperactive, disinhibited, had drooling of saliva and profuse sweating, generalized rigidity, and irritable affect. At 1300 hrs, he was found to have altered sensorium, mute, passed urine in bed, continued to sweat profusely, progressive lead pipe rigidity of whole body, pulse rate: 120/min, BP: 190/100 mmHg, body temperature 99°F, respiratory rate: 18/min, and was mildly dehydrated. He was clinically diagnosed to have developed NMS. All neuroleptics were withheld, blood investigations were sent and he was shifted to intensive care unit. Treatment was started with tab diazepam (5 mg), twice daily, bromocriptine (2.5 mg) thrice daily, trihexyphenidyl (2 mg) twice daily and other symptomatic measures. CPK was 1110 IU/L which rose to 4840 IU/L the next day and total leukocytes count became 12000 mm^3^. Other laboratory parameters and brain imaging were normal. From the fourth day of hospitalization, there was overall improvement, i.e. the sensorium improved, fever subsided, and rigidity reduced. CPK and TLC reached baseline by sixth day. In this period, his manic symptom reappeared and was managed with clonazepam (2 mg) thrice daily from third day and subsequently sodium valproate (1200 mg) and tab olanzapine (20 mg) in divided doses were added. There was no recurrence of NMS and manic features gradually subsided. He was managed as in-patient for 2 months.

### Case 3

A 23 yrs old male, again a case of manic episode, was hospitalized in the month of August 2004. He was treated in a peripheral hospital with tab haloperidol (5 mg) twice daily and inj haloperidol (5 mg) IM SOS for 3 days (total 4 inj were given). On admission, his treatment was continued with tab haloperidol (5 mg) thrice daily, tab trihexyphenidyl (2 mg) thrice daily, and tab sodium valproate (1200 mg) daily in divided doses. The patient developed fever of 100.3°F on fifth day along with tachycardia (116/min), diaphoresis, marked generalized rigidity, mild drowsiness, and restlessness. His blood pressure was 146/98 mmHg. All medicines were discontinued suspecting NMS. CPK was 964 IU/L and TLC was 10 900 mm^3^. He was managed in ICU with bromocriptine (2.5 mg) thrice daily and other symptomatic measures. Clinically, NMS subsided within 48hrs and manic symptoms reappeared. CPK became normal within 3 days.

## DISCUSSION

In this study, the prevalence of NMS was found to be quite low, only about 0.45%. All the three cases were of young males. All had acute and severe manic features and were given oral and injectable neuroleptics. NMS developed within few days of initiation of treatment with neuroleptics and subsided within few days of discontinuation of treatment.

Studies have suggested that NMS is more likely to occur in a patient who is in an agitated or dehydrated state, often needing restraint or who receives large doses of neuroleptic medication soon after admission to hospital and who continues to receive higher doses over next few days.[1,2,6,9–11] In our study, all the patients had these factors. Catatonic features have been described in patients who later developed NMS.[12–14] In our study, one patient had echolalia and echopraxia. Affective illness is another risk factor for NMS.[15–17] In this study, out of 672 psychiatric patients, 205 patients were suffered from mood disorders and all three patients reported were from this group. No patient from the remaining 467 non-affective group had NMS. Autonomic dysfunction is a core component of NMS,[17,18] which was present in our patients. Young males are reported to be more vulnerable for the development of NMS.[4] In our study, out of 672 male patients, only 42 were within the age of 20 years and two cases of NMS were in this age group. The other case was of 23 yrs old male. One interesting finding was that two cases occurred in summer months when atmospheric temperature was within the range of 38-44°C in day time and 28-32°C at night time. Such a variable is not reported earlier.

Patients may be more vulnerable to NMS during the hot summer months. One other interesting finding is that Case II had more severe symptoms but his fever was low grade as compared to Case I, who had less severe symptoms but higher temperature. Also olanzapine was used for treatment in Case II within 2 days of subsidence of his NMS, but it did not bring any relapse of symptoms of NMS, showing that it was safe.

For management of NMS a number of drugs have been tried and found to be effective. The drugs of choice are dantrolene (1 mg/kg/day) and bromocriptine (2.5 mg BD/ TDS upto 45 mg/ day). Other drugs which have been used are amantadine, levodopa, and benzodiazepines. Even ECT has been used effectively. Supportive measures like IV hydration, cooling blankets, ice packs, oxygenation, and antipyretics would also be required. Following recovery from NMS, one should avoid use of antipsychotics in the same patient. In case the use is unavoidable then only atypical antipsychotics should be used in lowest possible dosage keeping a watch for signs of NMS. Parentral depot preparations should not be used in these patients.[4]

Limitations of the study are as follows: Firstly, as almost all acutely psychotic patients were treated with antipsychotic medicines, no appropriate control group could be found. Secondly, as most of the patients were placed on antipsychotic medication early, it was difficult to determine whether any subtle sign was present before initiation of treatment. Thirdly, there was also the lack of blind conditions. Further studies may be able to overcome these shortcomings.
